# Fifteen-year follow-up of Italian families affected by arginine glycine amidinotransferase deficiency

**DOI:** 10.1186/s13023-017-0577-5

**Published:** 2017-02-02

**Authors:** Roberta Battini, M. Grazia Alessandrì, Claudia Casalini, Manuela Casarano, Michela Tosetti, Giovanni Cioni

**Affiliations:** 1 Department of Developmental Neuroscience, IRCCS Fondazione Stella Maris, Viale del Tirreno 331, Calambrone, 56128 Pisa, Italy; 2 Department of Developmental Neuroscience, MRI Laboratory, IRCCS Fondazione Stella Maris, Calambrone, Pisa, Italy; 3 Department of Clinical and Experimental Medicine, University of Pisa, Pisa, Italy

**Keywords:** Creatine synthesis defect, Magnetic resonance spectroscopy, Creatine supplementation, Longterm outcome, AGAT deficiency

## Abstract

**Background:**

Arginine:glycine amidinotransferase deficiency (AGAT-d) is a very rare inborn error of creatine synthesis mainly characterized by absence of brain Creatine (Cr) peak, intellectual disability, severe language impairment and behavioural disorder and susceptible to supplementary Cr treatment per *os*. Serial examinations by magnetic resonance spectroscopy are required to evaluate Cr recovery in brain during treatment of high doses of Cr per *os,* which have been proved beneficial and effective in treating main clinical symptoms.

A long term study with detailed reports on clinical, neurochemical and neuropsychological outcomes of the first Italian patients affected by AGAT-d here reported can represent a landmark in management of this disorder thus enhancing medical knowledge and clinical practice.

**Results:**

We have evaluated the long term effects of Cr supplementation management in four Italian patients affected by AGAT-d, correlating specific treatments with serial clinical, biochemical and magnetic resonance spectroscopy examinations as well as the neuropsychological outcome by standardized developmental scales. Consecutive MRS examinations have confirmed that Cr depletion in AGAT-d patients is reversible under Cr supplementation. Cr treatment is considered safe and well tolerated but side effects, including weight gain and kidney stones, have been reported.

**Conclusions:**

Early treatment prevents adverse developmental outcome, while patients diagnosed and treated at an older age showed partial but significant cognitive recovery with clear improvements in adaptive functioning.

## Background

**Fig. 1:**
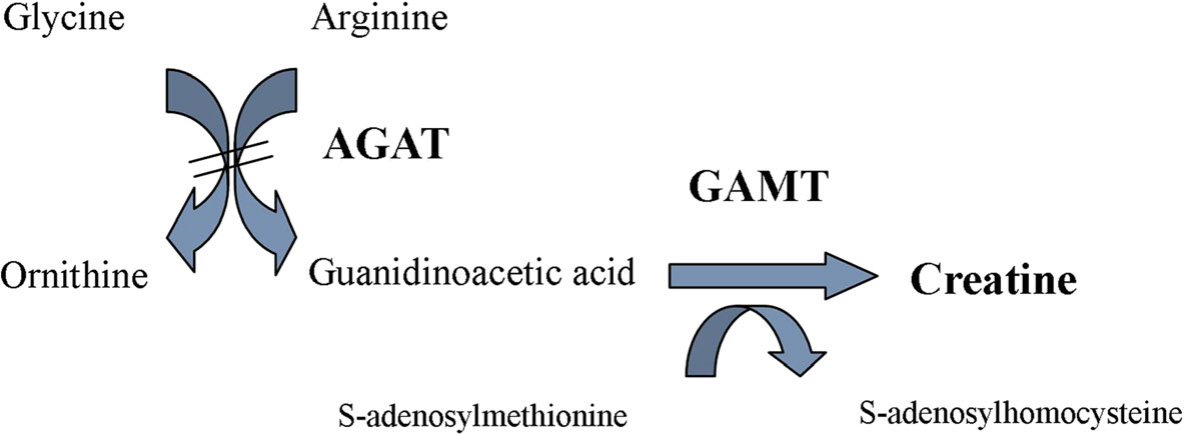
Metabolic pathway of creatine synthesis. Bars on the arrows indicate the blockage in AGAT-d

Arginine:glycine amidinotransferase (AGAT, OMIM 602360) deficiency (AGAT-d) is a very rare inborn error of creatine (Cr) synthesis described in 2000 in an Italian family and successively confirmed by molecular and enzymatic analysis [1, 2]. This disorder is caused by a deficiency of the first enzyme involved in Cr synthesis (Fig. 1), resulting in a commonly recognized biochemical pattern represented by low Guanidinoacetic acid (GAA) concentrations in plasma and urine and low/undetectable brain Cr detectable by magnetic resonance spectroscopy (MRS). Main clinical symptoms of this disorder are intellectual disability, severe language impairment and behavioural problems. Similar to guanidinoacetate-methyltransferase deficiency (GAMT-d), another Cr synthesis defect, it is susceptible of supplementation therapy with high doses of Cr monohydrate per *os* [3–6].

Since the first reports on two Italian families [1, 2, 6] 15 years ago, only 16 patients from eight families of different ethnic origins have been diagnosed [7].

There is a substantial lack of information on the outcome of these patients, due to recent detection of disease, limited number of years of substitutive treatment and paucity of identified patients. So far, a longitudinal 9-year study on a AGAT patient [8] and a recent review with some data on all AGAT-d patients identified worldwide [7] have been reported. In particular, this review confirms that the main clinical symptoms of the disease are present in all patients and reveals that myopathic symptoms are also present in about half of them, particularly in patients with a late diagnosis (from 6 to 23 years). Seizures and behavioural problems have also been reported in a few cases [7]. Chronic Cr supplementation of varying dosages, ranging from 100 to 800 mg/Kg/day, restores brain Cr to 60–95% of normal in all patients monitored by MRS, improving cognitive and motor symptoms [7], with the exception of one patient in which it increased only to 18% of normal even after 7 years of supplementation [7, 9]. Since blood and cerebral Cr concentrations during treatment have not been reported in all patients, it is not possible to establish a correlation, if any, between brain and blood levels. This information would be interesting in order to tailor treatments for each patient, thus avoiding high dosages and potential metabolic dysregulation. Therefore in this paper, we report the clinical data observed in our AGAT-d patients over a long period.

In fact, Cr treatment in these patients was pioneering and required constant adjustments according to clinical and biochemical evidence. During treatment, we attempted to answer the following questions which arose over time relative to patient outcome: a) which is the best dosage regimen? b) how long are brain Cr recovery times? c) how is neuropsychological impairment modified over time? d) is long term treatment safe for patients?

We updated previous data collected over time from the first Italian patients [1, 2, 6, 10–12] and we reviewed their long term outcomes in relation to clinical, neuropsychological, biochemical and toxicological findings after 10/15 years of Cr supplementation per *os*.

## Methods

### Study sample

Four patients from two related families are included in this report: two sisters (P1, P2, case index), diagnosed at 4 and 6 years respectively [1], and their brother (P4, neonatal diagnosis) [10] from family I and one male (P3) from family II, diagnosed at 2 years [6]. Their pedigree is already reported in our previous publication [6].

These subjects presented hyposomia, mild hypotonia, clumsiness, developmental delay with mild or moderate intellectual disability and severe language impairment [1, 6].

This research is part of a more general project on the treatment of primary Cr defects approved by the Stella Maris Institute Ethics Committee. Informed written consent was obtained from parents of each patient, before starting treatment trial with Cr per *os*.

DNA sequence analysis of *AGAT* was performed on each patient [2, 6, 10] and in order to understand better the role of enzymes involved in Cr metabolism disorders, a study of *GAMT* (OMIM 601240) was also performed on the two families [6].

Biochemical analyses of Cr and GAA in plasma and urine were performed in order to monitor Cr status in body fluids and therapeutic compliance of patients through follow-up. Galenic Cr-monohydrate supplementation per *os* in P1-P3 was initially administered at 400 mg/kg subdivided in 3 doses daily. After 6 years, this was reduced to 200 mg/kg for P1, P2 and to 300 mg/kg for P3 [11]. After about 10 years, it was further reduced to 100 mg/kg/die in P1, P2, P3, subdivided in two administrations. This dosage has been maintained until the present. Treatment of P4 started with Cr at 100 mg/Kg/day at 4 months of life [10] and remained substantially unchanged until the age of 10.

Since 2007, when the report on a 6-year outcome of our first 3 AGAT-d patients was published [11], all patients regularly underwent clinical (biochemical and neuropsychological) and MRS assessments, annually for P4 and every 18–24 months for the others.

### Clinical assessment

Clinical assessment consisted of monitoring: i) auxological data (weight, height and OFC); ii) routine laboratory evaluations (glycemia, BUN, creatininemia, hepatic enzymes, and urine examination); iii) Cr levels in blood and urine and plasma amino acids; iv) cognitive abilities by comprehensive neuropsychological evaluation.

In addition, considering the chronic high doses of Cr supplementation, creatinine and BUN were also initially evaluated every 6 months at home in order to monitor renal function.

Muscle strength was assessed after 11 and 15 years of follow-up by clinical grading scale (MRC scale).

Cr and GAA analysis in plasma and urine were performed by GC/MS [10] and plasma amino acids by an amino acid analyzer (JLC-500/V, Jeol, Japan).

Serial neuropsychological assessments were performed initially by means Griffiths Scales, Performance Scales of Wechsler Preschool and Primary Scale of Intelligence (WPPSI), visuomotor integration abilities test, language evaluation including productive and receptive vocabulary, sentence comprehension, and sentence repetition test [1, 6, 10]. Successively, all patients were monitored by Wechsler Intelligence Scale for children (WISC-r and WISC-III), Wechsler Adult Scale of Intelligence (WAIS-r) and WPPSI [12, 13]; in the last exam for P1-P3 by the Wechsler Adult Scale of Intelligence–IV Edition [12, 14] and for P4 by the Wechsler Intelligence Scale for Children-IV Edition [13]. These scales give an overall assessment of cognitive abilities for adults, young children and adolescents, respectively; providing a total score of Intelligence Quotient (TIQ) and four composite scores, which measure specific cognitive domains: Verbal Comprehension (VC), Visual-perceptual Reasoning (VPR), Working Memory (WM) and Processing Speed (PS). Adaptive functioning in motor, communication, daily living and socialization domains was assessed using the Vineland Adaptive Behaviour Scales (VABS) overtime [1, 6, 15].

### MRS assessment

1H-MRS was performed with 1.5 T clinical scanner (LX Signa Horizon 1.5 GE Healthcare, Milwaukee, WI, USA) and data were acquired from voxel (VOI) of 3.4 cc placed in parietal cortical gray matter [1, 6, 10]. Since 2007, we have monitored all patients also by 31P-MRS, performed in the same session using the same MR system operating at 25.86 MHz. Data were collected with a surface flexible spectroscopy coil placed around the head at maximum diameter, applying a hard pulse. Spectral width was 2500 Hz and 1024 complex data points were sampled with a TR of 4 s, a flip angle of 60 for a total of 128 averages [11, 16]. Phosphocreatine (PCr) and other high-energy phosphates such as inorganic phosphate (Pi) and nucleoside triphosphates (gamma-ATP, alfa-ATP and beta-ATP) were measured in terms of a ratio with respect to phosphodiester signal (PDE) [11]. PDE signal was chosen as internal reference since it demonstrated a steady signal amplitude in both normal subjects and in patients in this study and because this metabolite is not included in any known metabolic circuit for cellular ATP production. Spectra were processed with SAGE software package [11, 17]; pH was calculated from shift in resonance position of inorganic phosphate (Pi) peak compared to resonance position of PCr [11, 17].

## Results

### Clinical assessment

All patients showed a homozygous mutation c. 446 > A/p.Trp149* (stop mutation) in *AGAT* [2, 6, 10]. P1, P2 and P3 with their parents and 10 additional subjects of the pedigree were analysed by sequencing also *GAMT*. A sequence variation in exon 6 (T209M) at nt position 4024C > T of *GAMT* was disclosed, resulting in an amino acid change from threonine to methionine. The T209M sequence variation (dbSNP: rs 17851582) was carried heterozygously by 13 subjects and homozygously by 2 subjects (P1 and her asymptomatic father). However, normality of GAMT activity confirmed that this variation was non-pathogenic, without any clear consequences on enzyme protein function [6].

Plasma and urine Cr levels at diagnosis, after 5 years of Cr 200 mg/Kg/day (11-year follow-up) and 4 years of Cr 100 mg/Kg/day (15-year follow-up) for P1, P2, P3, and after 10-year follow-up for P4, respectively, are reported in Table 1. These data are different from those reported in the first papers on these patients [1, 6], probably due to the different techniques adopted for measuring low GAA and Cr concentrations. Cr levels increased under supplementation at all dosages compared to initial status, with highest concentrations at largest doses used. This was more evident in urine samples where very high levels of Cr, 4 to 10 times above the maximum reference value, were observed. Plasma Cr concentrations were otherwise within the normal range for dosages under 200 mg/kg/day.

**Table 1:** Plasma, urine and brain concentrations of Cr and GAA in AGAT-d patients at diagnosis and after different treatment

Cr dose (mg/kg/day)			T0				T1				T2			
	*GAA μM*		*Cr μM*		*Cr mM*	*PCr/PDE*	*Cr μM*		*Cr mM*	*PCr/PDE*	*Cr μM*		*Cr mM*	*PCr/PDE*
	P	U	P	U	Brain		P	U	Brain		P	U	Brain	
P1	n.d.	2.45	5.23	146	1.10	0.73	28.33	9,784	3.77	1.11	51.9	6,445	3.9	1.40
P2	n.d.	2.16	5.41	133	1.05	0.76	38.67	37,489	3.86	1.15	26.2	9,459	3.2	1.35
P3	0.10	1.34	4.59	138	1.33	0.76	136.0	44,736	3.56	0.86	31.5	756	3.5	0.87
P4^a^	0.13	0.54	16.22	25	0.15	n.a.	51.2	13,886	4.30	0.96	45.3	5,843	4.0	1.07
range:	0.22–3.14	56–698	18–141	200–5500	4.37+ 0.44	1.40+ 0.14								

*P* plasma, *U* urine, *n.d*. not detectable, *n.a*. not available

^a^Cr dose stable at 100 mg/kg/day overtime

Data were recorded at T0 (diagnosis), T1 (after 5 years of Cr 200, 300, 100 mg/Kg/day for P1-P2, P3 and P4, respectively) and at T2 (last observation at 100 mg/Kg/day for all patients)

Cr supplementation was reduced to 100 mg/kg/day in two administrations for P1, P2 and P3 after 11 years of treatment and have remained at this level until today, when a total amount of 3 g/day was adopted based on presumed daily need in humans [18].

Patient 4, supplemented with unchanged protocol since the first month of life (100 mg/kg/day), showed stable Cr levels in blood with a remarkable reduction in urine at the last exam (after 10 years of treatment), in line with the dose reduction due to weight gain, corresponding to about 60–70 mg/kg/day (Table 1).

Fasting plasma amino acids analysis showed normal results for all amino acids involved in Cr metabolic cycle which could be affected by long term Cr supplementation.

During chronic Cr supplementation, auxological data rapidly increased in all symptomatic patients, raising in less than one year from p 3–50 and to p 75–85 after 2 years of treatment. On the contrary, the asymptomatic patient showed normal growth since the first months of life, with similar increases after 2 years of treatment (about 4 years at age).

Weight gain in all patients increased to p 90–95 after few years of chronic Cr administration and have remained at this level until now, despite a low calorie diet.

**Fig. 2:**
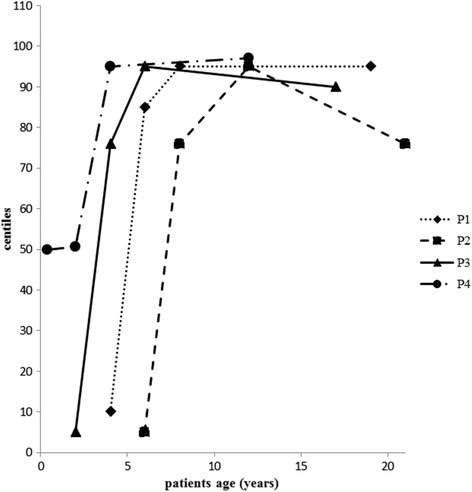
BMI changes in AGAT-d patients during Cr supplementation. Values higher than p 90 were observed in all patients in pre-pubertal age and have remained unchanged to the present

In all patients, high body mass indexes (BMI) were observed, especially in pre-pubertal age (Fig. 2), similarly for muscle bulk and muscle strength (grade 5 of MRC scale).

In the first years of therapy, patients presented polyuria and polydipsia. In addition, an occasional and transient diarrhea was observed when patients increased dosages to adjust for weight gain.

Routine laboratory evaluations were normal and patients did not show any other adverse effects.

Urinary sediment was also analyzed in all patients and none presented urine crystals, except for P3, who, at 10 years, had an episode of a kidney stone with no symptoms and spontaneously remittance after abundant hydration therapy. Daily urinary clearance and proteinuria were normal. P3 was thereafter monitored by renal ultrasound and urine examination every 6 months for three consecutive years, with no abnormal findings.

All patients have continued to participate in psychomotor and speech rehabilitation programs at home since diagnosis (2–3 times a week). They presented residual expressive speech problems, but achieved educational milestones in secondary school with special-education teachers, except for P4 who is now attending the fourth grade of primary school without any special aid and with a good profile. Educational and social level of family I, as well as dialect influences, could partially account for this problem. In addition, the environment of family II (divorced, poorly-educated parents and relatives, periods of incarceration of father and other factors), may have influenced compliance in supplementation and rehabilitation treatment and consequently cognitive development of P3.

**Fig. 3:**
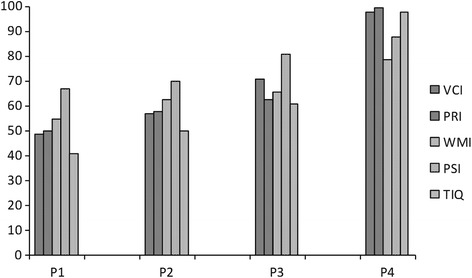
Psychometric evaluations by WAIS-IV or WISC-IV intelligence scales in AGAT-d patients after 15 years of clinical follow-up. Columns represent the scores obtained from each patient in different domains which contribute to the Total Intelligence Quotient (TIQ). Verbal Comprehension Index (VCI) and Visual-Perceptual Reasoning Index (PRI) in the asymptomatic patient were normal differently from his symptomatic relatives. Working Memory Index (WMI) and Processing Speed Index (PSI) in symptomatic patients presented better skills than the others

P1-P3 presented similar profiles on intelligence scales consisting of a greater fall in subtests designed to assess VC (VC Index, VCI) and nonverbal (VPR Index, VPRI) cognitive skills, and relatively better scores in subtests aimed at assessing WM (WM Index, WMI) and PS (PS Index, PSI). In fact, in all three patients, PSI statistically [14] differed from VCI and PRI and in P1 and P3 it also diverged from WMI. Cognitive difficulties of P1-P3 mainly affected processes of reasoning and knowledge building, both in verbal and nonverbal domains, while they showed better skills in working memory, attention, speed of execution and visual-motor integration (Fig. 3). A different profile was observed in P4, indicating only a slight fall in WMI, which was statistically lower than the others indices [13]. In comparison with normal TIQ of P4 (98), P1-P3 presented deficient scores (41, 50 and 61, respectively), with extensive falls in investigated areas, expression of intellectual disability which remained stable over years and was more severe in the older girl who started treatment after 6 years of age (Fig. 3).

However, P1 and P2, now 23 and 20 years respectively, obtained a high-school diploma: the oldest obtained a Hospitality Training Institute diploma and now works as a waitress in a pizzeria, while her younger sister earned a Biology Science Institute diploma and she is now studying with support for admission to a university bachelor program in biology. P3, now 18 years, is attending the last year of high-school (Hospitality Training Institute) with a lower profile than the other girls.

### MRS assessment

**Fig. 4:**
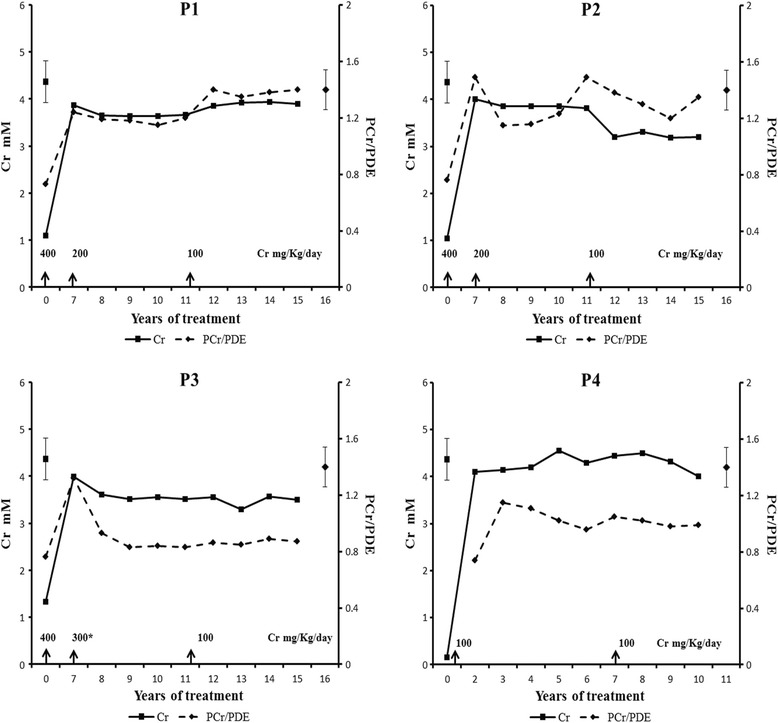
Brain Total Creatine (right axis, solid line) and PCr/PDE (left axis, dashed line) modifications recorded in the patients with different Cr amount. Normative data in the bars are represented as mean + SD

Consecutive MRS examinations have demonstrated that Cr depletion in AGAT-d patients is reversible under Cr supplementation. To our knowledge, only our patients were monitored by Proton (1H-) and Phosphorus (31P-) MRS in the same session during their long-term follow-up. Table 1 and Fig. 4 summarize the 1H-MRS and 31P-MRS data during follow-up.

Initial dosage of Cr supplementation was 400 mg/kg/day, corresponding to about 20 times daily Cr requirement for P1- P3, while P4 started his supplementation with a dose of 100 mg/kg/day. At 1H-MRS, an increase of Cr/PCr cerebral content was observed rapidly after supplementation, reaching over 90% in the three symptomatic subjects [1, 6], while in P4, Cr replenishment peaked at 60–65% [10]. Reducing Cr daily dose to 200 mg/kg in P1-P3 did not lead to any differences in tCr and PCr except for an unexpected drop in PCr/PDE ratio from 1.32 to 0.83 in P3 after an abrupt Cr reduction to 200 mg/kg/day. In order to avoid a further decrease of brain Cr, daily supplementation was immediately increased to 300 mg/kg/day [11].

During the following years, Cr daily supplementation was reduced to 100 mg/Kg in P1-P3 with no substantially differences in comparison to the highest doses (Table 1). A slight decrease in brain tCr peak was observed in P2 and P3 with a concomitant reduction in PCr peak compared to the results after 1 year at 200 mg/Kg/day [11, 12].

Brain Cr and PCr/PDE values in P4 showed an unchanged trend overtime, maintaining normal levels within 1 SD. Pi, ATP and pH in the brain were stable and within normal values in patients during all the above-mentioned treatments.

## Discussion

It has been well recognized that Cr supplementation is able to restore brain pool in patients with AGAT-d but unfortunately little information is available in terms of dosage, clinical progression and disease outcome. This study aimed at providing an update of results achieved in 4 AGAT-d patients after 15 (P1-P3) and 10 years (P4) of Cr supplementation, respectively.

Our experience tells us that the first critical step in diagnosis of AGAT-d consists of a proper quantitative measure of Cr and GAA in body fluids. As reported in the first paper by our group [1], misleading results could arise from urine samples, where levels of two key metabolites were near the lower end of normal value range, probably reflecting excretion of Cr assumed in food. Improvements in diagnostic techniques have eliminated this problem, and successive measurements in the same patients demonstrated that Cr and GAA levels in AGAT-d were below control range both in blood and urine indicating that they are, together with cerebral Cr levels, the best markers for AGAT-d.

The decision to set the initial dose at 400 mg/Kg/day in five administrations was based on an adopted schedule in a patient with GAMT deficiency, the first described inborn error of Cr metabolism [19], where 50% replenishment of brain Cr pool after 12 weeks of supplementation was reported. Similar brain Cr restoration was observed in our patients after 9 months of therapy, which resulted in 80% of normal [1] in gray matter and cerebellum, with concomitant improvements in cognitive development and other clinical symptoms.

Similar ameliorations have been observed in all the other patients [8, 9, 20]; for one of them the dosage was increased up to 800 mg/Kg/day and then reduced to 500 mg/kg/day without different replenishment of brain Cr, while progress in cognitive development was noticed after start of treatment [8].

Even though all patients showed clinical improvements under Cr supplementation; in patients where treatment started at a later age, an amelioration in muscle strength/endurance [9, 20] and in adaptive functioning [9] was limited, while cognitive and speech remained unchanged. Taken together, these two findings support the hypothesis that myopathy may be a clinical sign of disease only at an older age [7, 20] and that irreversible brain damage occurs when treatment is delayed. Our data seem to confirm this hypothesis because all patients diagnosed at a young age did not show muscle deficiency and cognitive recovery was lower for patients older at diagnosis. In fact, TIQ of P1 did not change despite brain Cr recovery. Conversely, early treatment prevents adverse developmental outcomes, as demonstrated in P4 [10, 12] and in the other young patient who started treatment at 16.5 months [8]; both of them, currently 11 and 12 years of life respectively, have achieved normal development with excellent educational milestones. According to previous assessments [12], WISC-IV score in the asymptomatic patient was within normal range, indicating that his intellectual development proceeded typically overtime with a slight residual speech impairment, also present to a greater degree in his sisters.

The placental Cr delivery to the foetus during pregnancy is important for an adequate growth and a development in normal conditions [21, 22], and crucial in Cr disorders, as demonstrated by the normal clinical presentation at birth of P4. Maternal supplementation during pregnancy could have provided higher levels of Cr in the affected newborn, but she refused any prior treatment as well as any prenatal diagnosis. In order to correct Cr depletion, since the infant was breastfed, we initially tried to supplement the mother’s diet with Cr. Although this approach resulted in an increase of Cr concentration in maternal milk, Cr increase in the infant’s blood, urine and brain was unremarkable [10].

Considering the clinical results in our 4 patients and taking into account the limitations of this study, some recommendations based on the evidence might be useful in the management of AGAT-d patients. Daily dose was reduced initially to 200 mg/kg/day in P1 and P2 after 6 years, and in P3 after 5 years, respectively, with good results [11]. Subsequently, based on MRS data obtained in the youngest patient, daily supplementation of the 3 older patients was gradually reduced to 100 mg/Kg/day with a parallel decrease of Cr levels in blood and urine, but not in the brain where Cr and PCr/PDE remained stable or decreased only slightly (Fig. 4). The slight reduction in brain Cr levels observed at the dose of 100 mg/Kg/day in these patients seems to have no effect on their cognitive performances, which are stable in the same range (Fig. 3), Indeed, in the asymptomatic newborn, in which the initial dosage was lower (100 mg/Kg), a 65% Cr restoration in brain required a longer period (about 18 months) than in his affected relatives, in which about 90% of Cr replenishment was observed after 12 months of therapy [1, 10]. Considering that the blood brain barrier is more permeable in developing brain and Cr was well absorbed in all patients, as demonstrated by Cr concentrations in plasma, the differences in brain recovery time observed in the 4 patients could be ascribed to the different dosage regimens adopted. Low doses require more time to restore Cr in brain to the highest possible levels without affecting clinical improvement, thus avoiding peripheral compartment overloading and possible toxic effects. These findings seem to be confirmed by data of the other young AGAT-d patient [8], in which doses higher than 400 mg/Kg did not improve Cr/NAA ratio more than 70% of controls and increased the risk of renal damage. In our patients, reduction of doses to less than 100 mg/kg/day normalized Cr in blood and urine, with the best profile in P4, who is biochemically similar to his normal peers.

1H MRS acquisition of P4 at the age of 8 years showed a slight reduction of cerebral Cr as in P3 at the same age, but not in the female patients, despite their different dosage regimens.

In our patients, 100% recovery of cerebral Cr seems unfeasible, whatever treatment schedule was used; the highest Cr replenishment was found in P4 at about 4 years (98%). Although timing dosages for all reported AGAT-d patients are incomplete, we suppose that the principal factor accounting for this effect is the down regulation of the transporter protein at high Cr concentrations [5, 23]. However, in the mouse model of AGAT-d, total Cr brain accumulation continues over time up to the normal concentrations of wild-type mice [24], while in AGAT-d patients brain Cr always remains below control values. Partial recovery of cerebral Cr in patients could represent a missing amount deriving from cerebral AGAT-d synthesis. Endogenous Cr synthesis contributes to brain needs for a negligible part (no more than 20%) as demonstrated by mouse models of Cr transporter (CrT) deficit [24, 25], where it is reduced in both the cortex and hippocampus to about 18–20% of control groups. In addition, brain Cr in CrT deficient patients is absent or strongly reduced when assessed by 1H-MRS; thus supporting the hypothesis that endogenous synthesis does not compensate for loss of Cr deriving from periphery. These considerations might account for the partial brain Cr replenishment in AGAT-d patients which does not reach the same level of controls and remains stable below 90% even when Cr transport is forced with high doses. However, despite the partial restoration of brain Cr pool, the clinical status of these patients improve significantly during treatment and, in the one who was early treated, typical somatic and psychomotor development was observed.

Considering the best dosage regimen, we can hypothesize that oral Cr supplementation efficiently restores cerebral levels even at lower doses, although more slowly, after a short period at high doses (400 mg/kg/day) in older patients or at 100 mg/kg/day in younger ones. The small number of patients included in this study and their common genetic background, affecting both the clinical course and treatment response, do not permit us to suggest clinical guidelines suitable for all patients with AGAT-d. However, as in other rare inborn errors of metabolism, each patient constitutes his/her own control and for this reason the optimal dosage treatment response is not an unquestionable statement. Furthermore, clear evidence for the effect of Cr on clinical improvement in AGAT-d patients arises from the observation that patient status normalizes during treatment.

## Conclusions

Despite the small number of patients, we conclude that 100% recovery of cerebral Cr seems unfeasible, whatever treatment schedule used, but there was no correlation between Cr levels and cognitive improvement. Cr treatment was well tolerated even at high doses, although side effects of weight gain and kidney stones were observed.

As indicated by the normal clinical development of P4, an early diagnosis is recommended for a timely treatment, suggesting the importance of NBS. Given the lack of a reliable biomarker in bloodspot for this inborn error of metabolism, it might be useful to consider other new options of treatment, such as untargeted metabolomics and/or genomic NBS. In the meantime, we should take in account AGAT-d in all patients presenting development delay, hypotonia, myopathy and language impairment in order to allow for an early diagnosis thus enabling treatment.

## Abbreviations

1H-MRS: Proton magnetic resonance spectroscopy
31P-MRS: Phosphorus magnetic resonance spectroscopy
AGAT: Arginine:glycine amidinotransferase
AGAT-d: Arginine:glycine amidinotransferase deficiency
BMI: Body mass index
BUN: Blood urea nitrogen
Cr: Creatine
CrT: Creatine transporter
GAA: Guanidinoacetic acid
GAMT: Guanidinoacetate-methyltransferase
GAMT-d: Guanidinoacetate-methyltransferase deficiency
GC/MS: GasChromatography/Mass-spectrometry
MRS: Magnetic resonance spectroscopy
NBS: Newborn screening
OFC: Occipito frontal circumference
PCr: PhosphoCreatine
PDE: Phosphodiester
Pi: Phosphorus inorganic
PS: Processing speed
TIQ: Total Intelligence quotient
VABS: Vineland adaptive behaviour scales
VC: Verbal comprehension
VOI: Voxel of Interest
VPR: Visual-perceptual reasoning
WAIS-r: Wechsler adult scale of intelligence
WISC-r and WISC-III: Wechsler intelligence scale for children
WM: Working memory
WPPSI: Wechsler preschool and primary scale of intelligence
